# Cardiac Sympathetic Neuromodulation in the Management of Refractory Electrical Storm: A Narrative Review

**DOI:** 10.3390/jcm15145540

**Published:** 2026-07-15

**Authors:** José M. López González, Daniel García Iglesias, Bárbara M. Jiménez Gómez, Luis Baeza, David Fernández Del Valle, Vanesa Alonso Fernández, Beatriz Díaz Molina, Marc Vives, José M. Rubín López

**Affiliations:** 1 Department of Anesthesiology, Perioperative and Pain Medicine, Central University Hospital of Asturias, 33011 Oviedo, Spain; josemanuel.lopezg@sespa.es (J.M.L.G.); barbarajimenezgomez@gmail.com (B.M.J.G.); luisbaeza93@gmail.com (L.B.); daviddelvalle92@hotmail.com (D.F.D.V.); 2 Department of Cardiology, Central University Hospital of Asturias, 33011 Oviedo, Spain; vanealfer@gmail.com (V.A.F.); beadimo@gmail.com (B.D.M.);; 3 Instituto de Investigación Sanitaria del Principado de Asturias (ISPA), 33011 Oviedo, Spain; 4 Department of Anesthesiology, Perioperative and Pain Medicine, University Clinic of Navarra, 31008 Pamplona, Spain; mvives@unav.es

**Keywords:** electrical storm, ventricular arrhythmias, neuromodulation, cardiac sympathetic denervation, stellate ganglion block

## Abstract

Electrical storm (ES) is a life-threatening clinical condition characterized by recurrent ventricular arrhythmias within a 24 h period, carrying a high mortality rate. Despite conventional therapies, including hemodynamic optimization, antiarrhythmic drugs, implantable cardioverter-defibrillator (ICD) reprogramming, and catheter ablation, a subset of patients develop refractory ventricular arrhythmias. In this setting, cardiac sympathetic neuromodulation can interrupt arrhythmic circuits by reducing efferent sympathetic outflow to the myocardium. Stellate ganglion block (SGB) with local anaesthetic (LA) is a temporary pharmacological blockade used as rescue therapy; in the largest prospective series (the STAR study), 92% of treated patients achieved at least a 50% reduction in arrhythmic events in the 12 h following the procedure. Because the effect of anaesthetic blockade is transient, more durable interventions have been explored, including percutaneous radiofrequency or chemical neurolysis and surgical cardiac sympathetic denervation (CSD), although current evidence is largely confined to small, uncontrolled case series. This narrative review synthesizes the available evidence on cardiac sympathetic neuromodulation—spanning SGB, percutaneous neurolysis, and surgical CSD—in refractory ES, positioning these interventions primarily as a means of stabilizing patients and bridging to definitive therapy rather than as established survival-modifying treatments. Furthermore, this review describes the primary anatomical foundations of the cervicothoracic sympathetic nervous system and the various techniques for SGB, along with their most relevant clinical indications. The risks and complications associated with these interventions are also addressed. Finally, clinical implications and potential future research directions in this field are discussed, with the aim of providing guidance for the comprehensive management of critically ill patients with refractory ES.

## 1. Introduction

Electrical storm (ES) is a life-threatening condition associated with high morbidity and mortality. It is defined as the occurrence of three or more episodes of sustained ventricular arrhythmia (VA) within a 24 h period, with episodes separated by at least 5 min and each requiring termination by anti-tachycardia pacing, cardioversion, or defibrillation. Incessant ventricular tachycardia, which is continuous or recurs promptly after termination, is a related but distinct entity considered separately. This clinical scenario occurs in 10–25% of patients with an implantable cardioverter-defibrillator (ICD) and is associated with a mortality rate of up to 14% within the first 48 h [1,2].

If left untreated, the short-term prognosis is fatal, leading to progressive and refractory heart failure [3]. Fundamental management includes hemodynamic optimization, the use of antiarrhythmic drugs (particularly amiodarone and beta-blockers), and ICD reprogramming to prioritize anti-tachycardia pacing (ATP) therapies. In selected cases, urgent catheter ablation is performed, yielding favorable immediate results [4].

Despite these well-structured and evidence-based treatments, VAs occasionally remain refractory. In such cases, neuromodulation reduces the efferent sympathetic tone to the myocardium, which may help suppress refractory arrhythmias. For some patients, this represents the final treatment option, whether as definitive therapy or as a bridge to transplantation. To achieve these results, complete blockade of the cervicothoracic sympathetic chain is required, which can be performed percutaneously through a stellate ganglion block (SGB) [5,6,7].

Although SGB has been shown to be effective in terminating ES within the first 24 h [8], robust clinical evidence regarding its long-term effects remains limited. To achieve a more durable cessation of cardiac adrenergic stimulation, more permanent neuromodulation techniques are currently available, including thermal radiofrequency cardiac sympathetic denervation (CSD), chemical denervation with neurolytic agents, and bilateral surgical cardiac sympathectomy. Current evidence is derived from small series reported in the literature [9,10,11,12,13]. However, the heterogeneity of these studies and the limited sample sizes necessitate further research to confirm these findings and establish standardized protocols for clinical management.

This narrative review provides a comprehensive overview of the current evidence regarding the efficacy of SGB in controlling refractory ES. It describes the most relevant anatomical considerations for performing the SGB technique and the neuromodulatory role of CSD, along with its associated risks and complications. Finally, clinical implications, limitations of the available evidence, and potential future research directions are discussed, aiming to guide the comprehensive management of critically ill patients with refractory ES.

## 2. Methods

This is a narrative review and did not follow a systematic search protocol. The authors identified relevant literature through non-systematic searches of PubMed/MEDLINE, Embase, Scopus, the Cochrane Library, and SciELO covering January 2000 to March 2026 (last search: 1 March 2026). Searches used free-text terms for the concepts “stellate ganglion block”, “cardiac sympathetic denervation/neuromodulation”, “radiofrequency/chemical neurolysis”, and “electrical storm/refractory ventricular arrhythmia”. Eligible study designs included original research articles, systematic reviews, meta-analyses, case series, and clinical guidelines published in English and reporting clinical, procedural, or safety outcomes of cardiac sympathetic neuromodulation. Because this is a narrative rather than a systematic review, no formal record-level selection flow (PRISMA diagram) was constructed; the sources most relevant to the scope of the review were selected by consensus among the authors, and a total of 62 references are cited in this review. Given the predominance of uncontrolled observational designs in this field, a formal meta-analysis was not performed; study quality was assessed narratively with attention to sample size, selection bias, absence of comparator groups, heterogeneity of arrhythmic substrates, and follow-up duration. The following is a structured evidence table (Table 1):

## 3. Pathophysiology of Electrical Storm

ES reflects a transient state of profound myocardial electrical instability in which triggers, a vulnerable substrate, and autonomic modulation converge. In most patients, the substrate is structural, myocardial scar from prior infarction or non-ischaemic cardiomyopathy, which creates zones of slow conduction and unidirectional block that favour re-entrant monomorphic ventricular tachycardia. Acute ischaemia, electrolyte disturbance (hypokalaemia, hypomagnesaemia), decompensated heart failure, pro-arrhythmic drugs, fever, and thyroid dysfunction act as triggers and frequently precipitate clustering [3,4].

Superimposed on this substrate, intense sympathetic activation is a central driver. Catecholamines increase intracellular calcium loading and the activity of the late sodium and L-type calcium currents, shorten and disperse refractoriness, and promote early and delayed after depolarisations; experimentally, left stellate ganglion stimulation lowers the ventricular fibrillation threshold. The resulting heterogeneity of repolarisation facilitates both triggered activity and re-entry, producing the polymorphic VT/VF and monomorphic VT that characterise ES. ICD shocks, pain, and anxiety further raise sympathetic tone, establishing a self-reinforcing cycle. This positive feedback between arrhythmia and adrenergic activation provides the mechanistic rationale for sympathetic neuromodulation: by reducing efferent sympathetic outflow to the ventricle, these interventions raise the fibrillation threshold and interrupt the cycle. Emerging strategies, including cervical vagal nerve stimulation and spinal cord stimulation, are under active investigation, though clinical evidence for the latter remains largely restricted to cardiac remodelling in heart failure rather than direct antiarrhythmic benefit in ES. In all cases, definitive therapy must be directed at the underlying substrate [20,21].

## 4. Anatomy of the Cervical and Thoracic Sympathetic Chain

The sympathetic nervous system is composed of preganglionic neurons, whose cell bodies are located in the brainstem and spinal cord, and postganglionic neurons situated in the paravertebral sympathetic ganglia along the laterovertebral chain. The sympathetic chain extends from the base of the skull to the coccyx, consisting of two bilateral paravertebral trunks containing segmental sympathetic ganglia, along with several prevertebral ganglia in the abdominal cavity (such as the celiac and superior/inferior mesenteric ganglia). The cervicothoracic portion of the sympathetic chain typically includes three cervical ganglia (superior, middle, and inferior) and the upper thoracic ganglia. The inferior cervical ganglion is frequently fused with the first thoracic ganglion, forming the cervicothoracic ganglion, commonly referred to as the stellate ganglion [22]. This ganglion provides sympathetic innervation to the head, neck, upper extremities, and upper thorax, including a significant contribution to the autonomic innervation of the heart.

Cardiac preganglionic sympathetic fibers primarily originate from the T1–T4 thoracic segments of the spinal cord and ascend through the cervical and thoracic sympathetic chain to innervate the heart. Each of the first thoracic ganglia (T1–T4) and the cervical ganglia sends efferent cardiac nerves that converge into the extrinsic cardiac plexuses surrounding the aorta and pulmonary arteries. Conversely, the vagus nerve provides cardiac parasympathetic innervation (mainly to the sinoatrial node and atria), establishing a modulatory balance with the sympathetic system. There is a specific topographical organization within this autonomic duality. Neuroanatomical studies suggest that the left atrium and left ventricle receive predominant sympathetic innervation, whereas the right atrium is under greater vagal influence. This distribution explains why sympathetic modulation (via left SGB) exerts a pronounced effect on ventricular electrical vulnerability, while the preservation of vagal tone may contribute to long-term arrhythmic stability [22].

The stellate ganglion is anatomically located anterior to the neck of the first rib and the transverse process of C7, immediately lateral to the longus colli muscle and deep to the prevertebral fascia. It is surrounded by several critical vascular structures: the carotid artery and internal jugular vein course anterolaterally, while the vertebral artery and inferior thyroid artery lie in close proximity. Additionally, it is closely related to the trachea medially and the esophagus on the left side. Inferiorly, the ganglion rests near the pleural dome. These anatomical relationships account for the risk of potential complications during the blockade procedure and justify the use of imaging guidance to ensure procedural safety [23].

In approximately 20% of individuals, the inferior cervical ganglion is not fused with T1 (absence of the classic stellate ganglion), implying that a block at the C7 level might not completely abolish sympathetic input from T1. Furthermore, anatomical variations such as Kuntz nerves, small accessory communicating branches from T2–T3 to the brachial plexus, may result in residual sympathetic innervation of the upper limb or even the heart following a standard SGB. These variants underscore the need to individualize denervation interventions and, in some cases, to consider extending the block to adjacent thoracic levels to achieve a more comprehensive effect [24].

## 5. Stellate Ganglion Block Techniques

**Figure 1:**
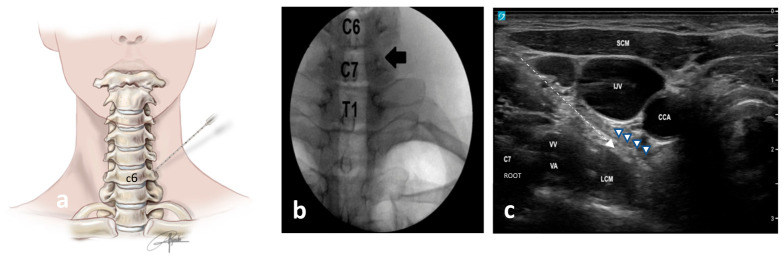
Techniques for stellate ganglion block. (**a**) Classic anterior (landmark-based) approach at the C6 level; (**b**) fluoroscopy-guided approach at the C7 level; (**c**) ultrasound-guided approach at the C7 level. The schematic illustrates needle trajectory relative to key structures and the position of the stellate ganglion deep to the prevertebral fascia over the longus colli muscle (arrowheads). Image created by the author of the study (JMLG). Original illustration (Figure 1a) used with permission from Alejandro García. Abbreviations: SCM, sternocleidomastoid muscle; IJV, internal jugular vein; CCA, common carotid artery; LCM, longus colli muscle; VV, vertebral vein; VA, vertebral artery; C7 root, C7 cervical root.

Several techniques have been described for performing an SGB, ranging from the classical landmark-based approach (also known as the “blind” block) to modern image-guided approaches using fluoroscopy or ultrasonography [25,26] (Figure 1). Historically, the blind block via an anterior neck approach was the original technique described by Leriche in the 1930s [27]. In this method, the patient is placed in a supine position with slight cervical extension. The anterior tubercle of C6 (Chassaignac’s tubercle) is identified as the key landmark, and the needle is inserted just lateral to the trachea to seek bony contact with the C6 transverse process before injecting the local anesthetic. While effective in experienced hands, this classical method carries a higher risk of inadvertent vascular or pleural puncture due to the lack of direct visualization of adjacent structures.

The introduction of fluoroscopic guidance significantly improved procedural precision [28]. This procedure is performed under anteroposterior radioscopic view, targeting the C7–T1 transition at the junction of the vertebral body and the transverse process. An oblique projection is then used to ensure that the needle tip is positioned anterior to the neural foramen. Contrast medium is injected to confirm adequate craniocaudal dispersion beneath the prevertebral fascia before administering the local anesthetic.

Ultrasound-guided blockade has become increasingly favoured. Ultrasonography enables clear identification of relevant anatomical structures, including the internal carotid artery, internal jugular vein, vertebral vessels, spinal roots, pleura, thyroid, and esophagus. It also allows precise localization of the stellate ganglion over the longus colli muscle, beneath the prevertebral fascia, with real-time needle tracking to reduce accidental injury [29]. Current consensus, however, permits ultrasound and/or fluoroscopic guidance, and the STAR protocol [14] incorporated both an anterior anatomical and a lateral ultrasound-guided approach; the choice of technique should reflect operator experience and local resources. Ultrasound-guided SGB can be performed at the C6, C7, or supraclavicular levels [30]; a C7-level injection is often preferred for ES because of broader coverage of the thoracic sympathetic chain, although this is not universally established.

For this approach, the transducer is placed at the C7 level to identify the vertebral body and the C7 nerve root exit, marked by the acoustic shadow of the posterior tubercle. The vertebral artery is typically located anterior to the C7 root. The stellate ganglion is found deep to the prevertebral fascia covering the longus colli muscle; its typical subfascial position is a critical consideration during infiltration. The use of color Doppler is essential to delineate the vertebral artery and thyroid vessels, preventing inadvertent puncture. Additionally, the position of the esophagus must be carefully monitored during a left-sided approach. Numerous studies have demonstrated that image guidance (ultrasound and fluoroscopy) reduces the risk of accidental intravascular injection and other complications compared to the blind technique [31]. Consequently, routine ultrasound guidance is recommended in current clinical practice for SGB, given its superior safety and efficacy profiles.

In the context of ES, SGB is usually performed on the left side, as left-sided sympathetic stimulation exerts a more potent pro-arrhythmic effect on the ventricles than right-sided stimulation. Nevertheless, if the response to a unilateral block is insufficient, a contralateral block may be considered to maximize suppression of cardiac sympathetic tone.

Currently, there is a lack of solid guidelines regarding the optimal pharmacological regimens and combinations of local anaesthetics (LA) for performing sympathetic blocks, although there is consensus that corticosteroids should not be used because they can cause damage to the central nervous system [32,33].

## 6. Clinical Indications for Stellate Ganglion Block

Traditionally, SGB has been utilized to treat conditions mediated by the sympathetic nervous system, such as complex regional pain syndrome (CRPS) and hyperhidrosis. However, recent evidence suggests that by transiently inhibiting sympathetic outflow, modulating regional vasodilation, and attenuating stress and inflammatory responses, SGB may serve as a safe and minimally invasive intervention for a variety of challenging clinical conditions [34,35].

The clinical scenarios in which SGB has demonstrated potential benefits are summarized in Table 2:

## 7. Sympathetic Neuromodulation in Refractory Electrical Storm

The influence of the sympathetic nervous system on ventricular fibrillation and malignant ventricular tachycardia is well established [36]. Stimulation of the stellate ganglion, particularly on the left side, decreases the ventricular fibrillation threshold and can trigger arrhythmias even in structurally normal hearts. Therefore, interfering with cardiac sympathetic innervation confers a protective antifibrillatory effect by preventing myocardial norepinephrine release. Based on this pathophysiological framework, sympathetic denervation interventions have been explored for decades to treat refractory arrhythmias. Beyond the extrinsic sympathetic chain, the intrinsic cardiac nervous system, including the intracardiac ganglionated plexi, is now recognized as an active processing network that modulates regional electrophysiology and arrhythmogenesis and represents an additional substrate for neuromodulation [17,22]. Within this autonomic framework, the sympathetic and parasympathetic limbs are functionally antagonistic, and cholinergic stimulation can blunt the arrhythmogenic effects of heightened adrenergic tone. Accordingly, low-intensity (weak) vagal stimulation, for example, non-invasive vagus nerve stimulation, has been proposed as a complementary, parasympathetically mediated strategy that might counterbalance the adrenergic surge driving an electrical storm, although clinical evidence in this acute setting remains preliminary and hypothesis-generating [37].

The classical approach is left cardiac sympathetic surgical denervation, such as the Wyatt or Schwartz procedures, which involve resection of the left stellate ganglion and the T2–T4 ganglia. This technique has demonstrated efficacy in reducing arrhythmias in hereditary syndromes and certain advanced cardiomyopathies [38]. However, in the acute setting of an ES, surgical availability is often limited; consequently, percutaneous SGB has emerged as a less invasive rescue alternative.

In the STAR study, the largest prospective multicentre experience to date, 92.2% of evaluable patients (those with treated arrhythmic episodes in the preceding 12 h) achieved at least a 50% reduction in treated arrhythmic events during the subsequent 12 h, with a median reduction of 100% (IQR −100% to −92.3%) and a single major complication (0.5%) [14]. These results should not be interpreted as complete arrhythmia suppression in all patients. Because STAR was an uncontrolled observational study, it can demonstrate a reduction in acute arrhythmic burden but cannot establish that SGB reduces mortality. This acute antiarrhythmic effect is attributed to its sympatholytic action, which reduces cardiac excitability and myocardial vulnerability to pro-arrhythmic stimuli. A 2024 systematic review and meta-analysis supports an acute reduction in arrhythmic events but remains limited by observational designs, heterogeneity, and short follow-up [19].

From an experimental perspective, studies in animal models have also shown that SGB increases the ventricular fibrillation threshold, highlighting the pivotal role of adrenergic surge in the heart’s electrical vulnerability and validating sympathetic neuromodulation as a therapeutic strategy in critical care contexts [39].

The antiarrhythmic effect of an anaesthetic block is, by nature, transient, and arrhythmias may recur once the block wears off if the underlying pro-arrhythmic state persists. SGB is therefore best regarded as a rescue or bridge therapy that buys time to deliver definitive treatment, most importantly catheter ablation of the arrhythmic substrate, escalation of mechanical circulatory support, or heart transplantation, rather than as a standalone solution. When a more durable effect is required, two routes are available. The first is surgical (thoracoscopic) cardiac sympathetic denervation (CSD), which in patients with inherited arrhythmia syndromes (notably long QT syndrome and catecholaminergic polymorphic ventricular tachycardia) reduces arrhythmia recurrence and is supported by comparatively robust data; its benefit in structural heart disease is less well established, and emergency surgery is often not feasible in haemodynamically unstable patients [18]. The second route comprises less invasive percutaneous techniques, radiofrequency or chemical neurolysis, developed to prolong denervation without surgery, but currently supported only by limited, uncontrolled data [15].

## 8. Clinical Implementation and Patient Selection

The incorporation of cardiac sympathetic neuromodulation into the management of refractory ES requires careful consideration of intervention timing, patient characteristics, and integration with other therapeutic modalities. Although temporary SGB and longer-lasting interventions (percutaneous radiofrequency or chemical neurolysis and surgical CSD) have shown promising reductions in ventricular arrhythmia burden, their optimal positioning within current treatment algorithms remains incompletely defined [2,4,19].

### Patient Selection and Predictors of Response

The identification of patients most likely to benefit from sympathetic neuromodulation remains an important unresolved issue. Available evidence suggests that treatment response may vary according to the underlying arrhythmogenic substrate and the relative contribution of adrenergic activation to arrhythmia initiation and maintenance [7,19].

It is useful to distinguish three broad substrates, which differ in their dependence on adrenergic drive and therefore in their expected response. First, scar-mediated monomorphic ventricular tachycardia, in which a fixed anatomical substrate predominates, and definitive substrate ablation is central, with neuromodulation acting mainly as a stabilizing adjunct [40]. Second, polymorphic VT or ventricular fibrillation during acute ischemia, in which the adrenergic contribution is high and sympathetic neuromodulation may be particularly useful [17]. Third, primary (inherited) electrical diseases, in which catecholaminergic triggering is variable. The evidence for left cardiac sympathetic denervation (LCSD) is considerably stronger in long QT syndrome and catecholaminergic polymorphic ventricular tachycardia, where adrenergic stimulation is a major trigger, than in other inherited conditions [38]. In particular, Brugada syndrome should not be grouped broadly with catecholamine-dependent syndromes, because its arrhythmogenesis is not primarily adrenergically driven and substrate (epicardial) ablation has a distinct and evolving role in this population [41]. Recently published multicentre experience also supports a role for sympathetic denervation in selected inherited cardiomyopathies, although the evidence remains preliminary [42].

Conversely, patients with advanced structural heart disease and extensive myocardial scar burden may experience less sustained responses. In these individuals, sympathetic denervation may suppress acute arrhythmic episodes but may not prevent long-term recurrence because the arrhythmogenic substrate remains present [43].

Several potential predictors of response have been proposed (Table 3):

Nevertheless, these observations derive predominantly from observational studies and small case series, and validated predictive models remain unavailable [7,19,44].

## 9. Radiofrequency Denervation of the Stellate Ganglion

Stellate ganglion radiofrequency (RF) denervation is a neuromodulation technique aimed at achieving thermal lesioning of the ganglion and adjacent cervicothoracic sympathetic fibers. This procedure is designed to produce a more durable denervation than that provided by local anesthetics, thereby interrupting the transmission of pro-arrhythmic stimuli from the sympathetic nervous system to the myocardium. This rationale is based on evidence that sympathetic hyperactivity persists once the anesthetic effect diminishes and is associated with increased ventricular arrhythmic burden and higher mortality; its modulation through sympathetic denervation can significantly reduce longer-term arrhythmic episodes and the frequency of ICD shocks [16,45,46].

Evidence for stellate ganglion RF in ES is limited to very small case series. These reports describe sustained suppression of ventricular arrhythmias and a reduction in ICD shocks during the acute period, with transient Horner’s syndrome being the most frequent minor side effect [15]. However, the major complication estimate of approximately 0.5% derives from percutaneous SGB procedures in the STAR study and should not be extrapolated to RF neurolysis, for which the true complication rate is unknown. Given the scarcity of data, percutaneous RF denervation should currently be regarded as an experimental or highly selected bailout strategy rather than an established therapy [47].

A further consideration is the possibility of bilateral intervention. Although the emphasis has traditionally been on left-sided treatment, some small series suggest that bilateral denervation might improve arrhythmic control in selected patients who do not respond to a unilateral approach. These observations are hypothesis-generating and derive from very limited, uncontrolled data; they do not constitute robust comparative evidence, and the proportion of non-responders who benefit from contralateral treatment cannot be reliably quantified at present [48].

The application of RF not only decreases the immediate need for electrical and pharmacological interventions but also facilitates patient stabilization during ES. However, long-term relapses still occur in a relevant proportion of cases, especially in patients with advanced cardiomyopathy. This suggests that even complete sympathetic denervation may not prevent long-term electrical instability if the underlying cardiac pathology progresses. Therefore, stellate ganglion RF should be integrated into a multimodal treatment approach rather than being considered a standalone solution. Once the patient is stabilized via sympathetic denervation, more definitive interventions should be evaluated, including endocardial ablation of arrhythmic substrates, maximal pharmacological optimization, and potentially heart transplantation [49].

## 10. Chemical Denervation of the Stellate Ganglion

Chemical denervation of the stellate ganglion using neurolytic agents, such as phenol (3–6%) or absolute alcohol (50%), has been described as an approach to achieve longer-lasting cardiac sympathetic denervation in patients with refractory ES. The supporting evidence relies heavily on historical phenol literature and isolated reports rather than on comparative studies; consequently, claims of efficacy comparable to radiofrequency cannot be substantiated. Chemical neurolysis should therefore be considered an experimental or highly selected bailout strategy, potentially useful for acute control of episodes resistant to other interventions but reserved for carefully selected patients [50].

Compared to other neuromodulation methods, chemical denervation is notable for its rapid onset of action. However, the duration of effect may be variable, and the available evidence remains limited. For this reason, chemical neurolysis should be considered within an integrated treatment pathway rather than as an isolated definitive therapy, and its use should be restricted to highly selected patients in whom the expected benefits outweigh the risks [40].

Chemical denervation has important limitations. The spread of the neurolytic agent is less controllable than thermal lesioning, which increases the risk of collateral injury to adjacent structures even when image guidance is used. A higher incidence of chronic adverse effects has been described with phenol compared to transient local anesthetic blocks. Potential complications include persistent Horner’s syndrome, upper-limb hypoesthesia, prolonged phrenic nerve palsy, and chronic neuropathic pain secondary to chemical neuritis [51]. Furthermore, the clinical effect of phenol may decrease over time due to cardiac reinnervation or neuroplasticity, meaning that arrhythmic control may not be permanent.

Consequently, chemical denervation is generally reserved for highly refractory patients in whom the potential benefits outweigh these substantial risks, for example, as an immediate bridge to heart transplantation in extreme cases. In these scenarios, strict radiologic monitoring of contrast medium dispersion is mandatory to limit the spread of the neurolytic agent.

It is noteworthy that refractory ES occurs most frequently in the context of advanced cardiomyopathy (e.g., extensive myocardial infarction or severe systolic dysfunction). In patients with irreversible ventricular impairment, sympathetic neuromodulation strategies may provide temporary stabilization and reduce the acute arrhythmic burden, allowing time for further treatment; however, heart transplantation may remain the only definitive therapy capable of restoring long-term electrical and hemodynamic stability. The initial success of sympathetic denervation must not delay the evaluation for transplantation or other definitive support measures, as neuromodulation does not address the underlying cardiac pathology [52].

Critically, while cardiac sympathetic neuromodulation shows promising results, the literature underscores the need for larger controlled trials to define standardized protocols, identify predictors of response, and accurately characterize the incidence of complications. This remains a significant challenge in consolidating its systematic use in refractory electrical storm [53].

The following table summarizes the main cardiac sympathetic neuromodulation techniques, distinguishing temporary blockade, percutaneous neurolysis, and surgical denervation, with mechanism, duration, guidance, and representative citations (Table 4):

We also added a table with relevant registered studies on the ClinicalTrials.gov registry (Table 5):

## 11. Complications and Clinical Considerations

As with any invasive intervention, sympathetic neuromodulation entails risks. In patients with refractory ES, the identification, prevention, and timely management of complications are critical, given their extreme physiological vulnerability. Generally, the safety profile of SGB is favorable. In the STAR study [14], only one major complication occurred among 184 procedures (0.5%), and the most common adverse effect was transient Horner’s syndrome (ipsilateral ptosis, miosis, and facial anhidrosis). Horner’s syndrome reflects cranial sympathetic spread of the injectate and is a marker of regional blockade; however, it should not be regarded as a reliable surrogate for complete cardiac sympatholysis, as the two do not necessarily coincide. This syndrome usually resolves spontaneously within hours or days without significant clinical consequences.

The most notable severe complication in temporary blocks is local anesthetic systemic toxicity (LAST), usually resulting from inadvertent intravascular injection, which can lead to seizures, malignant arrhythmias, or cardiovascular collapse. Fortunately, this risk is minimized by following strict puncture techniques, including frequent aspiration, fractionated dosing, and ultrasound guidance [56,57].

### 11.1. Local and Neurological Risks

Local risks include puncture site hematoma, infection, and direct injury to adjacent structures due to needle malposition (e.g., pleural puncture leading to pneumothorax or injury to the phrenic or cervical nerves). Most patients experience transient local discomfort that resolves within 48 h. Neurological complications such as dysphonia, due to recurrent laryngeal nerve block, and ipsilateral facial paresthesia, related to anesthetic diffusion toward cervical nerve roots, have been reported.

A rare but significant complication is hemidiaphragmatic paralysis caused by phrenic nerve involvement. In patients with limited respiratory reserve, an immobile hemidiaphragm can precipitate ventilatory failure; therefore, monitoring diaphragmatic function via ultrasound is recommended for high-risk individuals [58,59].

### 11.2. Risks Specific to Neurolysis and Radiofrequency

Neurolysis with phenol or alcohol carries additional specific risks. Phenol can cause tissue necrosis and fibrosing inflammation, which has been associated with permanent neurological deficits in a minority of patients. Ischemic vascular injury may also occur if the agent diffuses into the vertebral or carotid arteries. Compared to anesthetic blocks, neurolytic procedures have a higher incidence of chronic adverse effects, including prolonged phrenic palsy and persistent Horner’s syndrome.

Before performing RF denervation, coordination with the cardiology or device team regarding ICD management is essential [4]. Device management should be individualized rather than standardized. Suspension of tachytherapy is generally advisable during the use of electrocautery or RF energy to avoid electromagnetic interference and inappropriate shocks, and may be achieved by reprogramming or by applying a magnet over the device. It is important to note that, in most ICDs, a magnet inhibits tachytherapy detection but does not alter bradycardia (antibradycardia) pacing; asynchronous pacing is therefore not universally required and should be reserved for pacing-dependent patients, in whom reprogramming (rather than a magnet) is preferred. The grounding pad should be placed away from any metallic implants to prevent burns.

Before applying thermal energy, sensory and motor stimulation testing is mandatory to confirm that the needle is not in contact with the brachial plexus, phrenic nerve, or recurrent laryngeal nerve.

### 11.3. Contraindications

Contraindications must be weighed against the life-threatening nature of refractory ES. Patient refusal and infection at the puncture site are generally considered absolute contraindications. Systemic infection (sepsis) is best regarded as a relative rather than an absolute contraindication, given the theoretical risk of seeding a deep cervical space; notably, critically ill and septic patients were included in contemporary electrical-storm cohorts, including the STAR study [14], and underwent the procedure when clinically indicated. Coagulopathy and antithrombotic therapy are also relative contraindications. Because SGB is a deep cervical intervention near non-compressible vessels, decisions regarding anticoagulation and antiplatelet therapy should be individualized and aligned with regional anaesthesia anticoagulation guidance rather than relying on ultrasound guidance and compression alone. In the acute setting, the urgency of ES may nonetheless justify proceeding after a careful individualized risk–benefit assessment [60].

## 12. Discussion

The current body of evidence indicates that sympathetic nervous system modulation plays a pivotal role in managing refractory ES, while simultaneously highlighting significant limitations and ongoing challenges. In the acute phase (during the first hours to days after the procedure), the efficacy of SGB in suppressing malignant ventricular arrhythmias is well-documented in small series and case reports. These interventions successfully stabilize patients, acting as rescue therapy when antiarrhythmic drugs fail and providing an “arrhythmic pause” during which definitive treatments can be implemented [54,61]. However, the long-term sustained benefit remains less clear. Many patients experience recurrence of arrhythmias in the following weeks or months, especially if the underlying substrate is not addressed or if the primary cardiomyopathy continues to progress. Consequently, sympathetic denervation techniques should not be viewed as isolated solutions but as integral components of a stepped therapeutic algorithm. Ideally, after terminating the ES via neuromodulation, the patient should be evaluated for catheter substrate ablation, optimization of implantable devices, advanced heart failure therapies, or cardiac transplantation [49].

Definitive substrate ablation remains central: a recent network meta-analysis of randomized trials in ischaemic cardiomyopathy found catheter ablation superior to class III antiarrhythmic therapy for reducing appropriate ICD shocks and heart-failure exacerbations, with broadly comparable serious safety outcomes [62]. Neuromodulation is therefore best framed as a means of stabilising the patient until definitive therapy can be delivered.

A notable concern is the absence of large-scale randomized controlled trials and robust clinical guidelines supporting these therapies. To date, support for SGB and sympathetic denervation is derived from low-quality evidence, primarily observational series and case reports [15]. This lack of robust data explains why many arrhythmia management guidelines do not yet list sympathetic neuromodulation as standard of care, and currently, few centers possess the specialized expertise required to perform an emergency SGB. Formal incorporation of these interventions into clinical practice guidelines by cardiology societies will only be possible once more rigorous evidence is available.

There are also significant knowledge gaps regarding patient selection and treatment personalization. It remains unclear which patient profiles benefit most from sympathetic denervation or the optimal timing for implementation during refractory ES [55]. Furthermore, it is unknown whether a sequential combination of techniques, such as acute local anesthetic or neurolytic block followed by RF denervation, or unilateral followed by contralateral denervation, offers significant advantages over a single intervention. The heterogeneity of protocols reported in the literature makes it difficult to draw generalizable conclusions. A multidisciplinary and collaborative approach involving cardiologists, intensivists, surgeons, and anesthesiologists is required to develop dynamic algorithms that integrate the various therapeutic modalities available for the management of these critically ill patients.

Regarding procedural safety, sympathetic neuromodulation is generally a safe procedure with few complications, most of which are self-limiting or of minor clinical severity. Moreover, many of these complications are largely preventable. In this regard, thorough knowledge of the technique and the specific approach used in each case is essential, as the frequency of certain complications varies depending on the method. For instance, phenol use can be associated with Horner’s syndrome persisting for several weeks. Risks of local complications stemming from the close anatomical relationship of the stellate ganglion with structures such as the carotid artery and internal jugular vein can be significantly mitigated through image-guided approaches, particularly ultrasonography.

## 13. Conclusions

Cardiac sympathetic neuromodulation, whether temporary, through SGB, or longer-lasting, through percutaneous radiofrequency or chemical neurolysis or surgical CSD, is a useful adjunctive tool for interrupting refractory ES when standard measures have failed. The available evidence supports a reduction in short-term arrhythmic burden and the facilitation of definitive treatment, rather than an established survival benefit. When employed in a timely manner, it can stabilize critically ill patients and serve as a bridge to definitive therapies such as endocardial ablation of the arrhythmic substrate or cardiac transplantation in selected cases, reducing the immediate threat of recurrent lethal arrhythmias while the underlying substrate is addressed.

In this context, sympathetic blocks are becoming established as valuable rescue therapies. However, their effect is often transient and may be incomplete; thus, the recurrence of arrhythmias during follow-up remains a significant limitation. Consequently, these techniques must be integrated into a stepped, multimodal management strategy and should never be viewed as isolated interventions or as substitutes for other established therapies. They should be considered highly specialized procedures to be performed in experienced centers with careful patient selection.

## 14. Future Directions

Several key research avenues have been identified for future investigation. Multicenter randomized controlled trials directly comparing different sympathetic neuromodulation techniques are essential. These studies should evaluate the true incidence of complications, with particular focus on neurological and diaphragmatic function, while defining the optimal duration and ideal timing for implementation.

The development of dynamic algorithms that systematically integrate functional assessment tools and autonomic biomarkers could optimize candidate selection and the sequence of interventions. Furthermore, research into sequential or combined interventions involving sympathetic block, cardiac denervation, and bilateral approaches, alongside the exploration of emerging less-invasive technologies (such as non-invasive neuromodulation via focused ultrasound or extracorporeal shockwaves, non-invasive vagus nerve stimulation, and spinal cord stimulation), offers a promising avenue to improve clinical outcomes and reduce the mortality associated with refractory ES.

## Figures and Tables

**Table 1:** Summary of the principal clinical studies of cardiac sympathetic neuromodulation for refractory electrical storm and ventricular arrhythmias.

Study [Ref.]	Design	Patients (n)	Intervention	Main Outcome (Efficacy/Safety)	Quality (JBI)
Savastano 2024 [14]	Prospective multicentre observational (19 centres)	131 (184 procedures)	Percutaneous SGB	≥50% reduction in treated VAs in 92%; 1 major complication (0.5%)	Moderate
Tian 2019 [6]	Retrospective single-centre	30	Percutaneous SGB (left/bilateral)	60% free of VA at 24 h; no procedure-related complications	Low–moderate
Sanghai 2021 [5]	Retrospective comparative	18 (9 single, 9 continuous)	Left SGB: single vs. continuous infusion	VA reduction 94% (continuous) vs. 45% (single) over 24 h	Low–moderate
Millán 2023 [15]	Observational case series	7	US-guided SGB (±RF/surgical)	ES controlled in all; in-hospital mortality 14.3%	Low
Rao 2023 [16]	Prospective case series	6	Bilateral stellate ganglion RF ablation	All free of ES/VT at last follow-up; 2 appropriate shocks	Low
Vaseghi 2017 [17]	Multicentre (5 centres) retrospective	121	Surgical CSD (left/bilateral)	ICD shock-free survival ~50% at 1 y; mortality 25.6%	Moderate
Barwad 2021 [18]	Retrospective single-centre	65	Surgical CSD	>75% ICD-shock reduction in most; long-term benefit	Low–moderate
Meng [7]	Systematic review	38 (23 studies)	SGB in ES	Reduction in VA burden; low-quality evidence	n/a (SR)
Motazedian 2024 [19]	Systematic review + meta-analysis	542 (15 studies)	SGB in ES	Treated VAs reduced from 3.5 to 0 per patient	n/a (MA)

Abbreviations: CSD, cardiac sympathetic denervation; ES, electrical storm; ICD, implantable cardioverter-defibrillator; RF, radiofrequency; SGB, stellate ganglion block; SR/MA, systematic review/meta-analysis; VA, ventricular arrhythmia. Study quality was appraised with the Joanna Briggs Institute (JBI) critical appraisal checklist for case series; systematic reviews and meta-analyses were not scored with this tool. Overall, the evidence base is limited to observational designs (case series and retrospective cohorts) with no randomized controlled trials, small samples, heterogeneous arrhythmic substrates, and variable follow-up.

**Table 2:** Clinical indications for stellate ganglion block (SGB). The table summarizes the multidisciplinary applications of SGB, categorized by physiological system and clinical specialty.

Category	Indications
Cardiovascular	Refractory ventricular arrhythmias/electrical storm; refractory angina; cerebral vasospasm after subarachnoid haemorrhage.
Chronic pain/neurology	Complex regional pain syndrome (types I and II); post-herpetic neuralgia; sympathetically mediated head/neck/upper-limb pain.
Vascular	Raynaud’s phenomenon; acute limb ischaemia.
Other (established)	Severe primary craniofacial/upper-limb hyperhidrosis.

**Table 3:** Potential, unvalidated predictors of response to sympathetic neuromodulation. These are hypothesis-generating observations derived from observational studies and small case series, not validated predictors.

Potential, Unvalidated Predictors of Response to Sympathetic Neuromodulation
Predominance of polymorphic ventricular tachycardia or ventricular fibrillation.
High burden of recurrent ICD shocks.
Evidence of catecholamine-mediated arrhythmia triggers.
Limited myocardial scar burden.
Earlier intervention during the course of ES.
Lower degree of advanced heart failure progression.

Abbreviations: ICD, Implantable Cardioverter-Defibrillator; ES, Electrical Storm.

**Table 4:** Summary of the main cardiac sympathetic neuromodulation techniques, distinguishing between temporary block, percutaneous neurolysis, and surgical denervation, including mechanisms, duration, guidance methods, and representative references.

Technique	Mechanism/Agent	Duration	Guidance/Access	Representative Refs.
SGB—single injection	Local anaesthetic (e.g., bupivacaine/ropivacaine); reversible conduction block	Hours	Ultrasound and/or fluoroscopy; C6–C7 anterior/lateral	STAR [14]; Tian [6]; Fudim [8]
SGB—continuous catheter	Local anaesthetic infusion via indwelling catheter	Days (while infused)	Ultrasound-guided catheter placement	Sanghai [5]; Patel [13]; Dusi [54]
Percutaneous RF neurolysis	Thermal lesioning of ganglion/fibres	Weeks–months (variable)	Fluoroscopy ± ultrasound; sensory/motor testing	Rao [16]
Percutaneous chemical neurolysis	Phenol (3–6%) or alcohol (50%)	Variable; reinnervation possible	Strict imaging control of spread	Racz [50]
Thoracic epidural anaesthesia	Neuraxial local anaesthetic	Hours–days	Fluoroscopy/landmark; thoracic epidural	Lenarczyk [49]; Dusi [54]
Surgical CSD (VATS)—left or bilateral	Resection of lower stellate + T2–T4 ganglia	Permanent	Video-assisted thoracoscopic surgery	Schwartz [40]; Damiani [38]; Vaseghi [17]; Dusi [55];
Stellate-sparing LCSD	Resection sparing the stellate ganglion	Permanent (preliminary)	Video-assisted thoracoscopic surgery	Krieger [47]

Abbreviations: SGB, stellate ganglion block; RF, radiofrequency; CSD, cardiac sympathetic denervation; VATS, video-assisted thoracoscopic surgery; LCSD, left cardiac sympathetic denervation.

**Table 5:** Studies of cardiac sympathetic neuromodulation registered on ClinicalTrials.gov (accessed on 1 March 2026); registry status may lag actual study progress.

Registry ID	Design	Centre(s)	Intervention	Primary Endpoint	Status
NCT05720936	Multicentre observational	International (STAR study group, Italy)	Percutaneous SGB	≥50% reduction in treated arrhythmic events at 12 h	Completed; published 2024
NCT05078684	RCT, double-blind, sham-controlled	IKEM, Prague (Czech Republic)	Left SGB (bupivacaine) vs. sham	≥50% reduction in arrhythmic burden at 24 h	Ongoing (est. completion 2025)
NCT07211347	RCT	InCor, HC-FMUSP, São Paulo (Brazil)	SGB plus standard care vs. standard care	Reduction in VT/VF episodes and ICD shocks	Recruiting
NCT02646501	RCT	Yonsei University, Seoul (South Korea)	Percutaneous SGB vs. control	Effect on medically refractory VT	Unknown (last known: not yet recruiting)
NCT04043312	RCT, sham-controlled	Single-centre (USA)	Transcutaneous magnetic stimulation of the left stellate ganglion (non- invasive)	Reduction in VT burden	Completed
NCT05712122	Observational (retrospective)	Single-centre	US-guided left SGB (with corticosteroid)	Arrhythmic burden/ICD therapies	Completed
NCT07375264	Prospective single-centre observational	Central University Hospital of Asturias (Spain)	US-guided percutaneous bilateral CSD	Arrhythmic burden at 24 h and 12 months	Completed 2025 (unpublished)

Abbreviations: ID, identifiers; CSD, cardiac sympathetic denervation; ES, electrical storm; ICD, implantable cardioverter-defibrillator; RCT, randomized controlled trial; SGB, stellate ganglion block; VF, ventricular fibrillation; VT, ventricular tachycardia.

## Data Availability

No new data were created or analyzed in this study.
